# The Long Pentraxin PTX3 as a Link Between Innate Immunity, Tissue Remodeling, and Cancer

**DOI:** 10.3389/fimmu.2019.00712

**Published:** 2019-04-04

**Authors:** Andrea Doni, Matteo Stravalaci, Antonio Inforzato, Elena Magrini, Alberto Mantovani, Cecilia Garlanda, Barbara Bottazzi

**Affiliations:** ^1^Humanitas Clinical and Research Institute—IRCCS, Milan, Italy; ^2^Department of Biomedical Sciences, Humanitas University, Milan, Italy; ^3^The William Harvey Research Institute, Queen Mary University of London, London, United Kingdom

**Keywords:** pentraxins, PTX3, inflammation, tissue remodeling, wound healing

## Abstract

The innate immune system comprises a cellular and a humoral arm. Humoral pattern recognition molecules include complement components, collectins, ficolins, and pentraxins. These molecules are involved in innate immune responses by recognizing microbial moieties and damaged tissues, activating complement, exerting opsonic activity and facilitating phagocytosis, and regulating inflammation. The long pentraxin PTX3 is a prototypic humoral pattern recognition molecule that, in addition to providing defense against infectious agents, plays several functions in tissue repair and regulation of cancer-related inflammation. Characterization of the PTX3 molecular structure and biochemical properties, and insights into its interactome and multiple roles in tissue damage and remodeling support the view that microbial and matrix recognition are evolutionarily conserved functions of humoral innate immunity molecules.

## Introduction

Innate immune responses are the first strategies of host defense from invading pathogens and tissue damage. Their activation occurs when conserved structures on the surface of pathogens or associated with tissue damage, called pathogen associated molecular patterns (PAMPs) and damage-associated molecular patterns (DAMPs), respectively, are recognized by cell-associated or soluble molecules known as pattern recognition molecules (PRMs). Among soluble PRMs, pentraxins are a superfamily of evolutionarily conserved molecules with multi-functional roles in innate immunity and inflammation, such as regulation of complement activation and opsonization of pathogens (1). C-reactive protein (CRP) and serum amyloid P component (SAP) are the short or “classical” pentraxins. CRP is mainly produced by hepatocytes as an acute phase protein in man as well as other mammalian species, but not in mouse, in response to interleukin (IL)-6, whereas SAP is the short pentraxin acting as an acute phase protein in mouse (2). Pentraxin 3 (PTX3) is the prototype of the long pentraxin subfamily, originally identified as an IL-1 or TNF-inducible gene. PTX3 is produced by different cell types in response to primary pro-inflammatory stimuli and microbial moieties, is an essential mediator of innate resistance to selected pathogens of fungal, bacterial and viral origin [as discussed elsewhere (1, 3)], and is involved in regulation of inflammation, tissue remodeling, and cancer.

Here we will review the main biological features of PTX3 focusing on its structure and involvement in sterile conditions of tissue damage and cancer, and providing evidence that microbial and matrix recognition are evolutionarily conserved properties shared by humoral innate immunity molecules.

## Gene Regulation and Protein Structure

**Figure 1:**
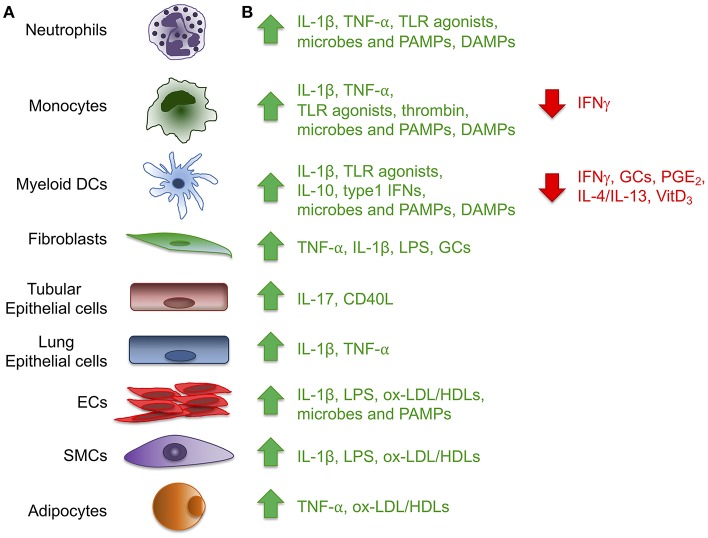
Expression of the long pentraxin PTX3. Several inflammatory stimuli, including positive (green) and negative (red) regulators **(A)** can induce PTX3 expression in different cell types, including cells of the myeloid lineage, fibroblasts, epithelial cells derived from different tissues, vascular and lymphatic endothelial cells (ECs), smooth muscle cells (SMCs) and adipocytes **(B)**. (CD40L, CD40 ligand; DAMPs, damaged-associated molecular patterns; GCs, glucocorticoids; LPS, Lipopolysaccharide; ox-LDL/HDL, oxidized-low-density lipoprotein/high-density lipoprotein; PAMPs, pathogen-associated molecular patterns; PGE_2_, prostaglandin E2; TLR, Toll like receptor; VitD_3_, vitamin D3).

The human and the murine PTX3 gene map on chromosome 3 and are organized in three exons, the first two coding for the leader peptide and the N-terminal domain, and the third coding for the C-terminal-pentraxin domain (Figure 1).

PTX3 is mainly induced by pro-inflammatory cytokines, such as IL-1β and TNFα, and by TLR agonists, microbial components (e.g., LPS, lipoarabinomannan, and outer membrane proteins of selected Gram-negative bacteria), and intact microorganisms (Figure 1). PTX3 expression is inducible in a wide variety of cell types, including fibroblasts and endothelial cells, myeloid cells such monocytes, macrophages, and dendritic cells (DCs), synovial cells, chondrocytes, adipocytes, glial and mesangial cells, epithelial cells and retinal cells (1, 4, 5) (Figure 1). High density and oxidized low density lipoproteins (HDL and ox-LDL) induce PTX3 production in endothelial cells and primary vascular smooth muscle cells (SMC) (6, 7). Microbial ligands stimulate the release of PTX3 from neutrophils, where the protein, mostly produced by myeloid precursors, is constitutively stored in specific granules (8, 9). Among peripheral blood mononuclear cells, only monocytes exposed to inflammatory cytokines or LPS produce PTX3 mRNA (1). PTX3 expression is negatively regulated by IFN-γ, IL-4, dexamethasone, 1α,25-dihydroxivitamin D3, and prostaglandin E2 (5, 10, 11). PTX3 is also induced by ovulatory stimuli in granulosa cells, and when released it contributes to the structural architecture of cumulus oophorus extracellular matrix (12).

PTX3 expression and production is regulated by different signaling pathways, mainly depending on the cell type and/ or stimuli. The NF-κB pathway controls PTX3 expression in conditions of IL-1 receptor- or TLR-dependent inflammation (13–15), while induction of the protein by TNFα in lung epithelial cells involves the c-Jun N-terminal kinase (JNK) pathway (16). HDL-induced PTX3 production in endothelial cells requires the activation of the PI3K/Akt pathway through G-coupled lysosphingolipid receptors (7).

**Figure 2:**
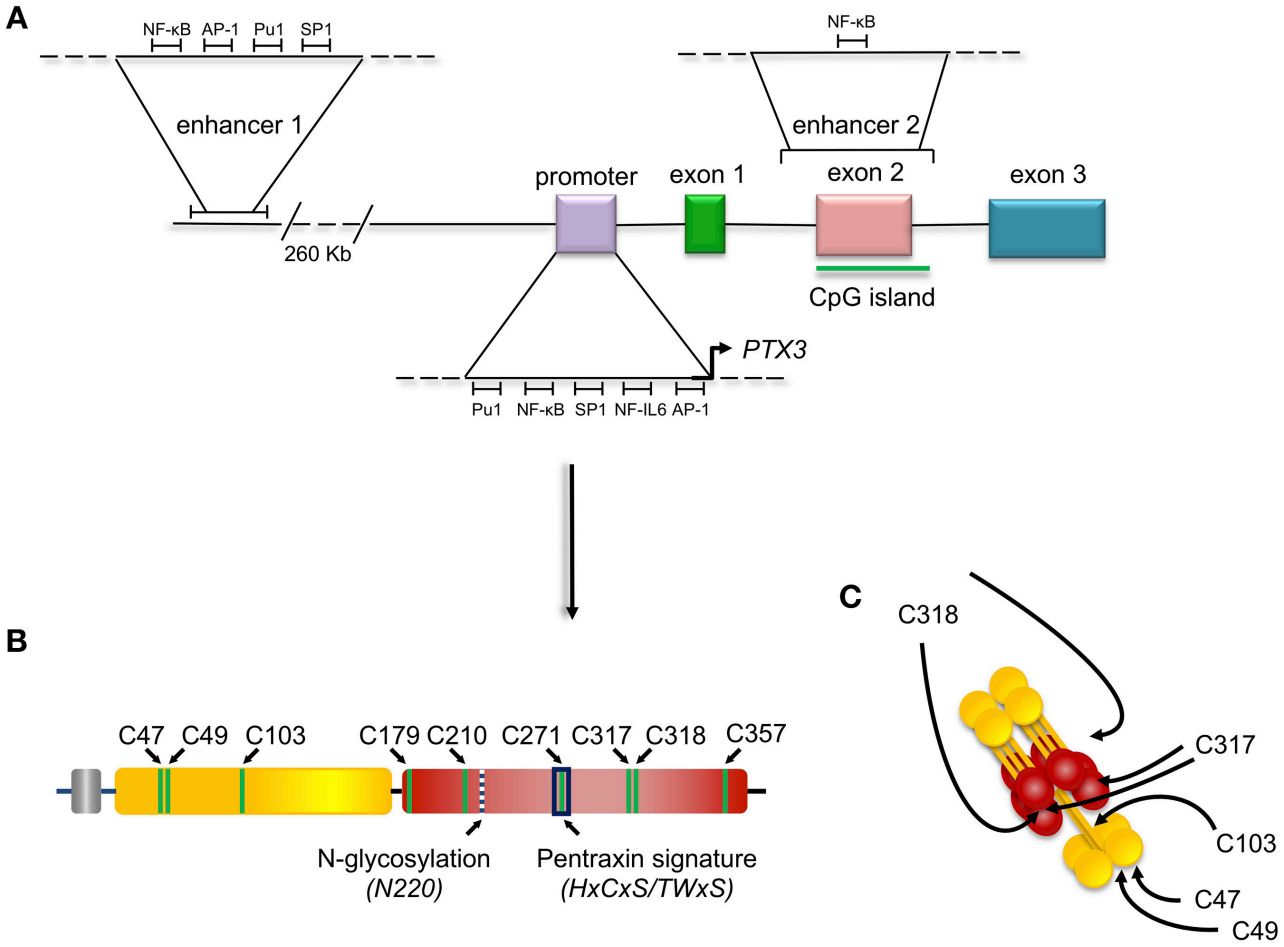
Gene and protein structure of the long pentraxin PTX3. PTX3 gene is located in chromosome 3 and is organized in three exons: the first coding for the signal peptide, the second coding for the N-terminal domain, and the third coding for the C-terminal, pentraxin domain. The promoter of PTX3 contains several transcription factor binding sites, including Pu1, NF-κB, SP1, NF-IL6, and AP-1. Depicted are also the sites of PTX3 epigenetic regulation, mediated by two potentially active enhancers. The first enhancer—containing the transcription factor binding sites for NF-κB, AP-1, Pu1, and SP1 - is located 230 kb upstream of the promoter, while the second enhancer—containing the transcription factor binding site for NF-κB—encompasses the second PTX3 exon **(A)**. Schematic representation of the PTX3 protomer subunit with the leader peptide in gray, the N-terminal region in yellow, and the pentraxin C-terminal domain in red. Shown are the Cys residues involved in intra- (C179-C357, and C210-C271) and inter- (C47-C47, C49-C49, C103-C103, C317/318-C317/318) chain disulfide bonds, the N-glycosylation site at Asn220, and the pentraxin signature (a primary sequence motif highly conserved across pentraxins) **(B)**. 8 protomer subunits assemble into an octameric protein stabilized by inter-chain disulfides (as well as non-covalent interactions), which are pointed to by arrows in the picture **(C)**.

The expression of the human PTX3 gene in physiological and inflammatory conditions is also regulated by epigenetic mechanisms. Hypermethylation of the promoter region and of an enhancer encompassing the second PTX3 exon (enhancer 2) (Figure 2) have been associated with PTX3 gene silencing in selected human tumors (e.g., colon rectal cancer and leiomyosarcoma) (14). Consistent with this, hypomethylation of these regulatory elements correlated with higher than normal protein levels in the plasma of coronary artery disease patients (17). Recent studies have characterized these epigenetic mechanisms in the context of different PTX3 expressing cells, including macrophages and fibroblasts, and have further addressed the epigenetic modifications occurring in the PTX3 gene in colorectal cancer (CRC) (18). These investigations identified a second enhancer located 230 kb upstream of the PTX3 gene promoter (enhancer 1, Figure 2). *In silico* and ChIP analysis revealed the binding of several transcription factors on this enhancer (18). Many of them, including the NF-κB subunit RelA, c-Jun, c-Fos, PU.1, and SP.1, are involved in the activation of inflammatory and immune responses, and are also known to control the activity of PTX3 promoter (Figure 2). The enhancer 2 was found only to bind NF-κB after TNF-α stimulation in macrophages, suggesting that this regulatory element could be important in the activation of tissue-specific transcription factors. However, the enhancer 2 could have a direct role in activating the expression of PTX3, since ChIP analysis showed its interaction with TAF1, a member of the transcription preinitiation complex (PIC) (18). Furthermore, STAT3-mediated hypermethylation of enhancer 1 has been associated with PTX3 gene silencing in colorectal cancers (18) (Figure 2). Interestingly, *in vitro* treatment of macrophages with glucocorticoid hormones, such as dexamethasone, results in M2 polarization, which is associated with immune suppression, and tumor progression (19). Noteworthy, one of the main markers of this phenotype is activation of STAT3, thus suggesting that STAT3-mediated PTX3 downregulation could be involved in carcinogenesis (see below).

Amongst PTX3 single nucleotide polymorphisms (SNPs), three (collectively forming an haplotypic block) have been found associated with susceptibility to infections including those caused by *Aspergillus fumigatus* (20–23), *Mycobacterium tuberculosis* (24), and *Pseudomonas aeruginosa* (25). Two of these SNPs are located in PTX3 intronic non-coding regions (rs2305619 in intron 1, and rs1840680 in intron 2), while the third is an exonic polymorphism that causes an amino acid substitution at position 48 (D48A, or rs3816527). Epidemiological studies have found a correlation between these three SNPs and PTX3 plasma levels, however the molecular mechanisms responsible for this association are still poorly understood. In this regard, individuals carrying the D48 allele have lower systemic concentrations of PTX3 (26). This might be due to faster rate of mRNA degradation, as proposed by Cunha et al. (20), or, alternatively, reduced activity of the second enhancer in the PTX3 gene (that encompasses the rs2305619, rs3816527, and rs1840680 SNPs) (18). It is not currently possible to exclude a direct local effect of amino acid substitution on protein structure (therefore function).

PTX3 is a multimeric glycoprotein whose protomer subunits comprise 381 amino acids. The protein primary sequence is highly conserved in evolution (with 82% identity between human and murine PTX3), likely due to early selection and enduring maintenance in phylogenesis of fundamental structure/function relationships. Analogous to other members of the long pentraxin sub-family, which includes guinea pig apexin, rat, human, and murine neuronal pentraxins 1 (NP1, or NPTX1) and 2 (NP2, also known as Narp or NPTX2), the putative integral membrane pentraxin NRP, and PTX4 (27), the PTX3 protomer is organized into an N-terminal region and a 203 amino acids long C-terminal domain with homology to the short pentraxins CRP and SAP (28) (Figure 2).

The N-terminal domain has no obvious similarity to any protein of known structure. However, secondary structure predictions indicate that this domain mostly comprises α-helical elements, three of which are likely organized into coiled-coils (6). Furthermore, the N-terminal end of this domain (amino acids 18–54) is predicted to be intrinsically disordered, a property that might provide the PTX3 protein with structural and functional versatility (29), thus contributing to the remarkable complexity of its interaction network (3).

The C-terminal domain shares with the short pentraxins a considerable degree of homology (with up to 57% similarity), which has allowed generation of 3D models based on the crystal structures of CRP (PDBID:1b09) and SAP (PDBID:1sac) (30–32) indicating that it adopts a β-jelly roll topology, stabilized by two intra-chain disulfide bonds (33). Two additional cysteine residues (i.e., Cys317 and Cys318) are involved both in intra- and inter-chain disulfides that, in conjunction with inter-chain bonds made by cysteine residues of the N-terminal domain, support the quaternary structure of the mature PTX3 protein (34) (Figure 2).

The pentraxin domain of PTX3 bears a single N-glycosylation site at Asn220 that, in a recombinant form of the protein from CHO cells, is fully occupied by complex type oligosaccharides, mainly fucosylated and sialylated biantennary sugars with a minor fraction of tri-and tetraantennary glycans. N-linked complex type glycosylation occurs in the natural protein made by human cells too (32), and mediates some of the PTX3 biological functions, including inhibition of influenza A virus hemagglutination (35, 36) and recognition of P-selectin (37). Furthermore, protein glycosylation (with major regard to sialylation) modulates the interaction of PTX3 with C1q, and the regulatory effect of PTX3 on complement activation via the classical pathway (32). We speculate that the molecular crosstalk between PTX3 and a range of diverse ligands involves a common glycan code, whereby tissue- and microenvironment-specific changes in the protein glycosylation profile might regulate its biological properties [see (38) for a review].

The modular (i.e., N- and C-domains) and sub-modular (i.e., coiled-coils and intrinsically disordered regions of the N-domain) nature of the protomer likely endows PTX3 with the structural versatility that is required to support its diverse interactions, thereby its biological functions. In this regard, the N-terminal region of the protein contains binding sites for fibroblast growth factor 2 (FGF2), inter-α-inhibitor (IαI), TNF-α-induced protein 6 (TNFAIP6 or TSG-6), plasminogen (Plg), fibrin, and conidia of *A. fumigatus* (15, 39–42). C1q and P-selectin mostly interact with the pentraxin-like domain (28, 37), whereas both domains have been implicated in the recognition of complement factor H (43, 44), and Ficolin-1 (45).

In addition to the multidomain organization, PTX3 has a complex quaternary structure with high-order oligomers stabilized by disulfide bonds. Mass spectrometry and site-directed mutagenesis indicate that PTX3 is made of covalent octamers (i.e., with a molecular mass of 340 kDa), through inter-chain disulfides bridges (34) (Figure 2). A low-resolution model based on data from electron microscopy and small angle X-ray scattering shows that eight PTX3 protomers fold into an elongated molecule with two differently sized domains interconnected by a stalk region, and a pseudo 4 fold symmetry along the longitudinal axis (33). Such quaternary structure is unique among pentraxins, where CRP and SAP both share a prototypical pentameric planar symmetry (46, 47). The only other pentraxin that forms an octamer is SAP from *Limulus polyphemus*, which, however, folds into a doubly stacked octameric ring (48). In addition, the oligomeric organization has important implications in its ligand binding properties. For example, the PTX3 octamer contains two binding sites for FGF2, and tetrameric recombinant forms of the N-terminal domain recapitulate the inhibitory functions of the full length protein toward this factor both in angiogenesis (33, 39) and bone deposition (49). However, dimeric forms of the N-terminal domain retain binding to IαI and TSG-6, thereby the octameric PTX3 protein is likely endowed with multiple (at least four) binding sites for each of these ligands, and can act as a nodal molecule in cross-linking hyaluronic acid in the extracellular matrix (41, 50).

High resolution models are urgently needed to disentangle the structural complexity of this long pentraxin and shed light on its structure/function relationships, some of which are remarkably different to those classically described for the short pentraxins.

## Role of PTX3 in Tissue Repair

Beyond its role as the first line of resistance against pathogens, innate immunity is involved in initiating the process of tissue repair (51–53). The cellular arm of the innate immune system senses specific DAMPs and regulates inflammatory responses at sites of damage (52, 54). The humoral arm of the innate immunity has different and complex roles ranging from the clearance of apoptotic cells and regulation of immune cell migration and activation, to regulation of remodeling cell activity (55, 56). For instance, SAP regulates fibrosis by inhibiting the alternative activation of macrophages via FcγRs (57) or by modulating immune cell activities via DC-SIGN (58). Pentraxins and components of the complement system also interact with elements present in the extracellular matrix (ECM), thus suggesting additional regulatory roles of the innate immune system in the tissue response to injury (53, 59). On the other hand, different ECM components, such as fibronectin, mindin, osteopontin, and vitronectin, interact with microbes and have opsonic activity (53, 66), thus suggesting a close evolutionary link between recognition of microbial moieties and ECM components.

**Figure 3:**
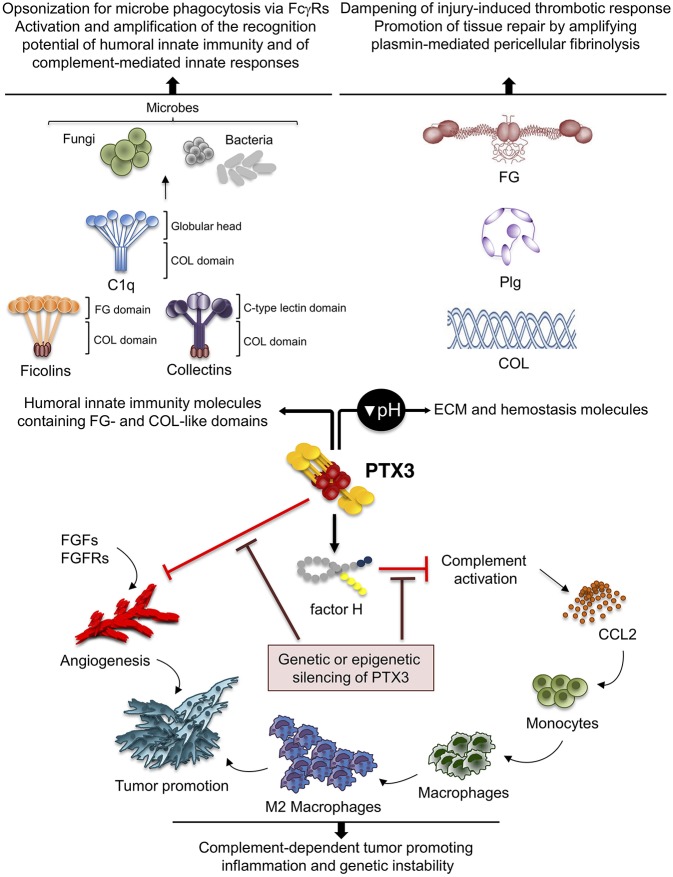
Role of PTX3 at the crossroad between innate immunity, hemostasis and tissue repair, and cancer. By interacting with fibrinogen (FG)- and collagen (COL)-like domains of other fluid-phase PRMs, such as C1q, ficolins, and collectins, PTX3 amplifies the recognition potential of these members of the humoral innate immunity, favoring complement-mediated antimicrobial resistance, and effector functions (upper left panel). By interacting at acidic pH with fibrinogen/fibrin and collagen, as well as plasminogen (Plg), PTX3 tunes injury-induced thrombotic responses and favors pericellular fibrinolysis, contributing to tissue remodeling and repair (upper right panel). By interacting with factor H and FGFs, PTX3 controls complement-dependent tumor promoting inflammation, including CCL2-dependent macrophage recruitment and M2-like skewing, as well as FGF-dependent neo-angiogenesis. This anti-tumor potential of PTX3 is hampered by genetic or epigenetic silencing of PTX3 leading to enhanced tumor growth (lower panel).

In different mouse models of non-infectious tissue damage, deficiency of the long pentraxin PTX3 was associated with altered thrombotic response to the lesion, increased deposition and persistence of fibrin, followed by increased collagen deposition (15, 62, 67–69) (Figure 3).

Following tissue damage, PTX3 was induced in the blood and locally in response to TLR activation and IL-1β amplification (15). Interestingly, PTX3 is reported to be among the genes induced by thrombin in monocytes (70). At sites of wound, PTX3, released by neutrophils (9), localized in the clot and in the pericellular matrix of macrophages and PDGFRα^+^FAP^+^ cells of mesenchymal origin that collectively invade the wound site (15, 68).

In skin wounding, PTX3-deficiency was associated with increased deposition of fibrin, followed by increased deposition of collagen, fibroplasia, epithelial hyperplasia, and delayed healing (15). A premature contraction of the wound was observed in PTX3-deficient mice, in agreement with an augmented content of platelet-derived factors (e.g., thrombin, serotonin, PDGF, TGFβ) known to be responsible of skin wound contraction by SMC located in the *panniculus carnosus* (71–73). Indeed, administration of pharmacological inhibitors of coagulation and platelet activation reverted these defects, including premature wound contraction and increased collagen deposition (15). Therefore, an altered haemostatic and fibrinolytic response triggered the alterations associated with PTX3-deficiency in skin wound healing.

In CCl_4_-induced liver injury, PTX3 was localized in necroinflammatory areas and fibrotic portal tracts, and was associated with neutrophils, macrophages and mesenchymal stromal cells (MSCs) (15). In this setting, PTX3-deficiency was associated with increased centrilobular thrombosis and fibrin deposition in necroinflammatory areas, followed by severe impairment of repair and fibrosis, as assessed by increased α-SMA^+^ fibroblastic cells and collagen deposition (15). Similar abnormalities were reported in different models of lung injury (15, 74). In addition, PTX3 played a protective role in a murine model of ischemic injury of the brain, where it was involved in the resolution of edema and glial scar formation (75). PTX3 administration reverted the IL-6/STAT3-dependent interstitial fibrosis in a mouse model of acute kidney injury (76).

Fibrin is deposited after tissue injury and its subsequent timely removal is essential for several aspects of tissue repair in major organs, as well as in a wide range of pathological conditions (77, 78). In these contexts, a defective fibrinolysis is described as an etiopathological factor leading to reduced remodeling and altered connective tissue formation (79–81). Macrophages and MSCs enter the wound site invading the inflammatory matrix through plasmin-mediated mechanisms, allowing fibrin removal and consequent deposition of granulation tissue rich in type I collagen, as well as other ECM proteins (71, 81, 82). The alterations in tissue repair observed in PTX3-deficient mice have been attributed to defective plasmin-mediated invasion and fibrinolysis by tissue remodeling cells, namely macrophages and MSCs (15, 68). Also *in vitro*, PTX3-deficient macrophages and fibroblasts showed defective fibrinolytic activity (15), thus suggesting that PTX3 contributes to the progression of a normal and efficient pericellular fibrinolysis which promotes repair.

PTX3 was shown to interact specifically with Plg and fibrin at acidic pH (optimal range from 6.5 to 5.5), but not at neutral pH (15) (Figure 3). Interestingly, the interaction of PTX3 with members of the ficolin and collectin family, occurring through their fibrinogen-like domain and collagen domains, is facilitated in an acidic microenvironment (45, 83, 84). Acidification of the wound site, which occurs as a result of cellular metabolic adaptation to trauma-induced tissue hypoperfusion, has functional relevance in the healing outcome and involves several processes including cell adhesion, migration, and proliferation (53, 85). The interaction of PTX3 with fibrin and Plg occurs through different sites in its N-terminal domain and PTX3 does not interfere with the interaction between fibrin and Plg. In mapping experiments, PTX3 did not interact with the Plg Kringle 1 domain, indispensable for Plg initial recruitment on lysine-rich portions of fibrin and/or on cell surface, but specifically bound the Kringle 5 domain (15). This interaction could be crucial for triggering Plg conformational changes that allow a transition of the molecule from a closed-inactive to an open and functional form (86–88). This conformational transition is essential for Plg conversion into plasmin operated by Plg activators (PAs) and central in fibrin removal in the thrombus (78, 89). Indeed, in cell-free fibrinolysis assays, the interaction of PTX3 with Plg determined the enhancement of plasmin-mediated fibrin gel degradation triggered by urokinase PA (uPA) and tissue-type plasminogen activator (tPA) at acidic, but not neutral, pH. Plg activators are neutral proteases (90). The dependence on acidic pH of the interaction of PTX3 with fibrin and Plg ensures that it does not occur in the circulation but rather at sites of tissue repair and in thrombi, where it supports fibrinolysis in acidic environments. Thus, the acidic pH acts as a “switch on” signal for this function of PTX3 (Figure 3). The interaction with fibrin and Plg is restricted to PTX3, since no similar function has been reported for short pentraxins. Only one study reported the interaction of SAP with fibrin and consequent modulation of *in vitro* formation of clots (91), however the underlying molecular mechanism has never been characterized.

In a model of arterial thrombosis, PTX3 produced by the vessel wall had a critical protective role in the modulation of thrombus formation (62). Fibrinogen pre-incubation with PTX3 significantly reduced platelet aggregation in the presence of collagen. Likewise, pre-incubation of collagen with PTX3 attenuated platelet aggregation in the presence of fibrinogen. These effects were dependent, respectively, on the N-terminal or C-terminal domain of PTX3, and suggested that in arterial thrombosis PTX3 disfavored the pro-thrombotic activity of fibrinogen and collagen (62). PTX3 interacts with P-selectin and tunes P-selectin-dependent neutrophil extravasation (37). However, in arterial thrombosis PTX3 did not influence P-selectin-dependent platelet-leukocyte and platelet-endothelium aggregation (62, 92). Although initially PTX3 has been reported to induce tissue factor (TF) expression in endothelial cells and monocytes (93), subsequent *in vitro* and *in vivo* studies did not confirm this result (15, 62). Indeed, in the thrombosis model TF expression in the aorta of PTX3-deficient mice and controls was similar (62). These results are in line with the evidence that PTX3 plays protective functions in vascular pathologies. Indeed, PTX3 overexpression limited the neointimal thickening after rat carotid artery balloon injury (94) and PTX3-deficiency was associated with augmented infarct area following myocardial ischemia/reperfusion injury (13), increased atherosclerosis and augmented macrophage accumulation and inflammation in atherosclerotic plaques (95).

The administration of MSCs to acute or chronic wounds improves wound healing by increasing granulation tissue formation, accelerating re-epithelialization and stimulating angiogenesis through paracrine signaling (96), thus prompting new studies on the treatment of non-healing wounds resulting from burns (97) and Crohn's disease (98). In wounded skin, MSCs acted as a potent promoter of tissue repair and remodeling, whereas PTX3-deficient MSCs showed compromised recruitment and invasiveness at the site of damage, due to defective fibrinolysis, and therefore exerted a compromised therapeutic effect causing delayed healing (68).

Similar results were obtained in a mouse model of acid aspiration-dependent acute lung injury (69), mimicking acute respiratory distress syndrome (ARDS) caused by aspiration of gastric contents (99). In the mouse model, beneficial effects of treatment with MSCs on the early acute inflammatory reaction, pulmonary edema and long-term fibrotic evolution and pulmonary function have been observed. The administration of PTX3-deficient MSCs was less effective in limiting the pulmonary edema at 24 h after acid aspiration, and was associated with defective fibrinolytic activity, resulting at later time points in increased pulmonary fibrosis and therefore in a not significant increase of lung function. Levels of D-dimer significantly increased in mice after treatment with MSCs indicating their ability to modulate pulmonary fibrinolysis and thus affecting fibrotic scarring. The administration of PTX3-deficient MSCs resulted in decreased lung levels of D-dimer compared to PTX3-competent MSCs, thus attributing to a defective fibrinolysis the observed reduced therapeutic effects of PTX3-deficient MSCs (69).

Recently, PTX3 has been identified as an important molecule contributing to bone homeostasis and remodeling (49). Under homoeostatic conditions, histological analysis of distal femurs of PTX3-deficient mice did not show differences in the number of active trabecular and endosteal TRAP^+^ osteoclasts. However, micro-computed tomography showed a lower bone volume attributable to suppression of the osteoblast function. In a fracture and regeneration model of the tibia diaphysis, PTX3-deficient mice showed a lower bone formation and repair rate than controls, in agreement with lower percentage of mineralized callous tissue and lower collagen I expression compared to controls. Under conditions of homeostasis and bone repair, the expression of PTX3 was associated with non-hematopoietic/non-endothelial periosteal cells, in particular, with CD51^+^ and α-SMA^+^ osteoprogenitor subsets. FGF2 is expressed during the early stages of bone formation and is abundantly accumulated in the bone matrix, where it participates in osteoblastogenesis and skeletal remodeling (100). In agreement with the property of PTX3 to bind FGF2 and prevent FGF2-dependent activities, PTX3 reversed the negative effect of FGF2 on osteoblast differentiation from bone marrow stromal cells *in vitro*, and the PTX3 N-terminal domain alone recapitulated this activity. Therefore, PTX3 produced by osteoblast lineage cells, acts as a bone protective factor, important to unlock osteoblast maturation by antagonizing the FGF2 effect during bone formation (49). Bone formation during fracture repair initiates around extravascular deposits of fibrin-rich matrix and subsequent defects in fibrin clearance from the fracture site severely impair healing (101). Fibrinogen depletion in Plg-deficient animals restores a normal fracture repair (102), thus proving that inefficient fibrin turnover is essential for bone repair. Therefore, further studies are needed to address the relevance of PTX3-dependent modulation of the fibrinolytic system in bone repair.

All together, these studies have provided several lines of evidence that the involvement of PTX3 in tissue remodeling and repair depends on the recognition of matrix molecules and highlight the connection and interplay between haemostasis and immunity (Figure 3).

## Role of PTX3 in Cancer

Inflammation is a component of the tumor microenvironment promoting tumor development and growth (103). Since PTX3 is expressed in inflammatory conditions and acts as a tuner of complement-activation and leukocyte recruitment, it was hypothesized that PTX3 was involved in cancer-related inflammation. Genetic studies in mice showed that PTX3-deficiency caused increased susceptibility to mesenchymal and epithelial carcinogenesis in the models of 3-methylcholanthrene (3-MCA)-induced sarcomagenesis, and 7,12-dimethylbenz [a] anthracene/terephthalic acid (DMBA/TPA)-induced skin carcinogenesis (14). In these tumors, infiltrating macrophages and endothelial cells were the major source of PTX3 in response to locally produced IL-1. PTX3-deficient tumors were characterized by increased macrophage infiltration, pro-inflammatory cytokine production, complement activation, and angiogenesis, as well as increased oxidative DNA damage and genetic instability, compared to wild type tumors (14). In this context, PTX3 regulated complement activation by interacting with factor H, a complement regulator, and as a consequence, macrophage recruitment and M2-like polarization (14) (Figure 3).

These data are in line with recent studies showing that the anaphylatoxins C3a and C5a may contribute to cancer-related inflammation, recruit myeloid suppressor cells, and promote IL-1β and IL-17 response in neutrophils thus promoting colon carcinogenesis (104–107).

In addition to regulate complement, PTX3 was shown to bind selected fibroblast growth factors (FGFs), including FGF2, and FGF8b through the N-terminal domain, and inhibit FGF-dependent angiogenic responses (6). This effect was shown to be responsible of the anti-tumor activity of PTX3 in FGF-dependent transplanted murine tumors, including prostate cancer and melanoma and fibrosarcoma (108–110) (Figure 3). The role of PTX3-mediated anti-angiogenic activity has not been addressed so far in primary carcinogenesis.

In line with these preclinical studies, the human PTX3 promoter and regulatory regions were shown to be epigenetically modified through hypermethylation in selected human mesenchymal and epithelial cancers, such as esophageal squamous cell carcinoma (111) and colorectal cancer (14, 18, 112), leading to silencing of PTX3 protein expression. Thus, genetic studies in mice and epigenetic studies in humans demonstrate that PTX3 behaves as an extrinsic oncosuppressor gene by acting at the level of complement-mediated, macrophage-sustained, tumor promoting inflammation.

In contrast to the genetic evidence outlined above, several studies performed with PTX3 overexpressing cells suggest that the protein may play a pro-tumorigenic role by promoting tumor cell migration and invasion (head and neck tumors, cervical cancer) or proliferation (glioma), epithelial-mesenchymal transition (hepatocellular carcinoma) and macrophage chemotaxis (64, 65, 113, 114). In basal-like breast cancers, PTX3 was found to be a critical target of oncogenic phosphoinositide 3-kinase signaling and NF-κB-dependent pathways, and to be associated with PI3K-induced stem cell-like traits (115). However, none of these pro-tumoral effects of PTX3 has been confirmed in gene targeted animals or in carcinogenesis models.

These contradictory results suggest that PTX3 may have a dual role in cancer, likely depending on the type of cancer, or on the cells producing it, in particular tumor cells or infiltrating macrophages, fibroblasts and endothelial cells. Further genetic studies in mice and humans will be necessary to clarify these context-dependent findings.

## PTX3 as Marker of Cancer Progression

Several lines of evidence indicate that PTX3 could be a local or systemic marker of cancer-related inflammation. Upregulation of PTX3 gene expression was observed associated to a stromal signature in ovarian cancer (116), and has been described in aggressive breast cancer and distant bone metastases (117–119), anaplastic thyroid carcinoma (120), soft tissue liposarcoma (121), prostate cancer (122), and glioblastoma (123). Increased circulating levels of PTX3 were observed in myeloproliferative neoplasms (124), soft tissue sarcomas (125), lung cancers (126–128), pancreatic carcinomas (129), gliomas (130), and hepatocellular carcinomas (131). In pancreatic carcinoma, high PTX3 levels were associated with advanced clinical stage and poor overall survival. In the same cohort of patients with invasive ductal pancreatic carcinoma at stage III and IV, plasmatic CRP levels were similarly associated with a worst prognosis (129).

Different studies analyzed the role of PTX3 as biomarker in lung cancer. Through a proteomic effort, Planque et al. reported in 2009 that PTX3 is produced by lung cancer cells. This result was confirmed in patients with lung cancer, in which PTX3 plasma levels resulted significantly increased compared to healthy subjects (126). It was subsequently observed that PTX3 circulating levels were related to disease aggressiveness and progression, irrespective to the subtypes and histotypes of lung cancer (127). In addition, ROC analysis indicated that PTX3 could discriminate between cancer patients and heavy smokers at high risk for lung cancer (127). Similarly, high PTX3 levels were correlated with worse progression-free survival in patients with lung cancer and chronic obstructive pulmonary disease (132), and with overall survival and disease-free survival in small-cell lung carcinoma (SCLC) (133). A recent study on 1358 individuals at high risk for lung cancer demonstrated that PTX3 levels were not predictive of pathology occurrence (128). In the 110 patients of this cohort that developed resectable lung cancer, preoperative PTX3 plasma levels were higher compared with those of cancer-free heavy smokers, but were not predictor of outcomes (128).

In prostate cancer patients, circulating levels of PTX3 were higher compared to patients with prostatic inflammation, while serum levels of prostate-specific antigen (PSA) and CRP were not different between the two groups (134). In CRC, PTX3 circulating levels were significantly increased compared to healthy individuals or to patients with colorectal polyps, representing an independent prognostic factor for CRC patients (135). PTX3 levels were reduced at discharge after surgery, and a subsequent increase during the follow-up was associated to recurrence. Preoperative PTX3 levels were significantly associated to clinical stage and to a better postoperative prognosis in a cohort of 263 primary CRC patients (136). In another small group of CRC patients, PTX3 serum levels combined with CXCL8 and VEGF levels were efficiently predicting relapsing cases (137). Since epigenetic studies showed PTX3 silencing in colorectal tumor cells (18), increased PTX3 plasma levels in these patients reasonably reflect cancer-related inflammation associated with tumor growth. Patients with hepatocellular carcinoma (HCC) showed higher PTX3 levels than individuals with fibrosis (131). Interestingly, in these patients the A/A genotype for rs1840680 and rs2305619, resulting in higher PTX3 plasma levels, was also significantly associated with the presence of HCC.

Beside an evaluation of PTX3 as soluble biomarker in cancer, some reports also investigated PTX3 expression in cancer tissues. In hepatocellular carcinoma, PTX3 expression was analyzed after liver resection in tumoral and adjacent normal tissue and a higher PTX3 expression was observed in the tumoral area. PTX3 expression was correlated with advanced stage, larger tumor size, presence of intra-hepatic metastases, portal vein tumor thrombosis and liver cirrhosis (65). Overall, high PTX3 expression in tumor tissue from HCC was associated with lower survival after surgery. Immunohistochemical analysis on tissue specimens from lung cancer revealed an interstitial expression of PTX3 in the neoplastic area associated with shorter survival, while no staining was observed in normal lung parenchyma (128). In tissue samples from prostate cancer patients, PTX3 is expressed at higher levels compared to patients with prostatic inflammation (134).

Overall the data reported above strongly suggest that PTX3 is overexpressed locally or systemically in different neoplastic conditions, and could likely represent a novel promising prognostic factor for cancer patients. In particular, as discussed above and by Giacomini et al. (63), PTX3 originated from endothelial cells, tumor-associated fibroblasts and infiltrating myeloid cells likely reflects microenvironment or systemic inflammation associated with tumor progression, and not its involvement in the pathogenesis. Indeed, the role of PTX3 in neoplastic transformation and growth has been shown to depend on the context and to be influenced by its property to interact with different molecules in the tumor environment.

## Concluding Remarks

Based on genetic studies in mice and human genetic associations, PTX3 is a well-recognized mediator of innate resistance to selected infections, acting by modulating complement activation, opsonizing microbes and facilitating their clearance through phagocytosis. Moreover, by interacting with the fibrinogen-like and collagen-like domains of ficolins and collectins, PTX3 amplifies the recognition potential of the humoral innate immunity (1) (Figure 3). These lines of evidence provide the rational for therapeutic and diagnostic translation of this molecule in infectious conditions.

Several studies presented in this review also indicate that PTX3 is involved in tissue remodeling and repair in sterile conditions through the recognition of matrix molecules, and regulates the thrombotic response and fibrin remodeling, thus playing a non-redundant role in the orchestration of the tissue repair process (Figure 3). Other humoral PRMs interact with ECM components (e.g., C1q, collectins, CRP, SAP), or contain collagen- and fibrinogen-like domains (e.g., ficolins, MBL, collectins), and several ECM molecules recognize microbial moieties and have opsonic activity (e.g., fibronectin, mindin, osteopontin, vitronectin). These lines of evidence support the view that inflammation, innate immunity, haemostasis, and tissue repair are functionally linked and that the recognition of microbial moieties and extracellular matrix molecules by the humoral arm of innate immunity is evolutionarily conserved.

Studies reported here finally show that PTX3 is involved in tuning carcinogenesis through the modulation of cancer-related inflammation or angiogenesis in specific cancer types (Figure 3). However, other studies propose that in specific models PTX3 has a pro-tumorigenic function, by promoting tumor cell migration and invasion and macrophage infiltration, suggesting that PTX3 may have different functions on carcinogenesis depending on the tissue and cancer type, and possibly cell- and stimulus-dependent PTX3 glycosylation (and sialylation) profiles, which needs further dissection.

Cancer is considered a “non-healing wound” (138), since wound-healing responses favoring tumor growth are activated in the tumor microenvironment. These include extravascular deposition of fibrin that acts as a provisional stroma for stromal and immune cells migration, angiogenesis and ECM deposition and remodeling (139). Fibrin degradation, vascular resorption and collagen synthesis result in formation of dense fibrous connective tissue (“scar” in wounds and “desmoplasia” in cancer). These responses are similar in tumors and wounds, but in tumors they are not self-limited. PTX3 by interacting with fibrin matrix (15), FGF2 (109), and complement components (14) regulates the main common processes in tissue repair (139–141) and in tumor-promoting angiogenesis and inflammation (Table 1) (61, 142, 143), thus suggesting that the roles of PTX3 in tissue repair and cancer are functionally associated.

**Table 1:** Biological functions of PTX3 in tissue remodeling and cancer.

		**Ligands**	**Functions**
Tissue remodeling	Fertility	Hyaluronic acid/TSG-6/inter-α-trypsin inhibitor	Incorporation of PTX3 into the hyaluronic acid-rich ECM surrounding the pre-ovulatory oocyte (i.e., dependent on the presence of IαI and TSG-6) is essential for cumulus matrix stability and female fertility (12)
	Synaptogenesis	ND	Pentraxin 3 regulates synaptic function by inducing AMPA receptor clustering via ECM remodeling and beta1-integrin (60)
	Bone turn-over	FGF2	PTX3 is expressed by osteoblast progenitors, and is essential for matrix mineralization both in bone tissue homeostasis and fracture repair (49)
	Angiogenesis	FGFs	PTX3 recognizes selected FGFs via its N-terminal domain, and inhibits their binding to FGF receptors, thus preventing endothelial/smooth muscle cell proliferation *in vitro* and angiogenesis/neointima formation *in vivo* (61)
	Fibrinolysis	Fibrinogen/fibrin/plasminogen	PTX3 derived from macrophages and mesenchymal cells forms a tripartite PTX3/fibrin/plasminogen complex at acidic pH that promotes pericellular fibrinolysis (15) In a mouse model of arterial thrombosis, PTX3 inhibits platelet adhesion and aggregation by targeting fibrinogen and collagen (62)
Cancer	Anti-tumoral	factor H	In murine models of chemically induced mesenchymal and epithelial carcinogenesis, PTX3 dampens cancer-related, complement-dependent inflammation (14)
		FGFs	PTX3 inhibits the FGF-driven tumor cell proliferation *in vitro*, tumor growth, angiogenesis and metastatic potential *in vivo* in models of melanoma, prostate, breast and lung cancer (63)
	Pro-tumoral	Not defined	PTX3 promotes tumor cell migration, invasion and metastasis, and protein levels correlate with prognosis and/or tumor grade in different types of cancer (64, 65)

## Author Contributions

CG and BB revised the manuscript and redacted the final version. AD, MS, AI, EM, AM, CG, and BB contributed to the writing of the manuscript.

### Conflict of Interest Statement

The authors declare that the research was conducted in the absence of any commercial or financial relationships that could be construed as a potential conflict of interest.
